# Logistic feasibility of health related quality of life measurement in clinical practice: results of a prospective study in a large population of chronic liver patients

**DOI:** 10.1186/1477-7525-6-97

**Published:** 2008-11-10

**Authors:** Jolie J Gutteling, Jan JV Busschbach, Robert A de Man, Anne-Sophie E Darlington

**Affiliations:** 1 Department of Gastroenterology and Hepatology, Erasmus MC, 's Gravendijkwal 230, 3015 CE, Rotterdam, the Netherlands; 2 Department of Medical Psychology and Psychotherapy, Erasmus MC, Dr. Molewaterplein 50, 3015 GE Rotterdam, the Netherlands

## Abstract

**Background:**

The objective of the present study was to provide a complete and detailed report of technical and logistical feasibility problems with the implementation of routine computerized HRQoL measurement at a busy outpatient department of Hepatology that can serve as a tool for future researchers interested in the procedure.

**Methods:**

Practical feasibility was assessed by observing problems encountered during the development of the computer program, observing patients' ability to complete the HRQoL questionnaires, monitoring the number of times that patients completed the HRQoL questionnaires and observing logistics at the outpatient department. Patients' reasons for not completing the HRQoL questionnaires were assessed retrospectively by means of a mailed questionnaire. Physicians' attitudes towards the availability of computerized HRQoL information about their patients were assessed by means semi-structured interviews and by means of checklists administered after each consultation with s study participant.

**Results:**

All physicians (n = 11) and 587 patients agreed to participate in the study. Practical feasibility problems concerned complicated technical aspects of developing a user-friendly computer program and safe data transmission over the Internet, patients' lack of basic computer skills and patients' lack of compliance (completion of questionnaires on only 43% of the occasions). The main reason given for non-compliance was simply forgetting, which seemed to be related to reception employees' passive attitude towards sending patients to the computer. Physicians were generally positive about the instant computerized availability of HRQoL information. They requested the information in 92% of the consultations and found the information useful in 45% of the consultations, especially when it provided them with new information.

**Conclusion:**

This study was among the first to implement the complete procedure of routine computerized HRQoL measurements in clinical practice and to subsequently describe the feasibility issues encountered. It was shown that the attitudes of physicians were generally positive. Several barriers towards successful implementation of such a procedure were encountered, and subsequently solutions were provided. Most importantly, when implementing routine computerized HRQoL measurements in clinical practice, assistance of an IT professional for the development of a tailor-made computer program, availability of questionnaires in multiple languages and the use of touch-screen computers to optimise patient participation are essential. Also, all staff of the department concerned should approve of the intervention and consider it as part of standard clinical routine if successful implementation is to be obtained.

## Background

The importance of patients' health related quality of life (HRQoL) in medical practice is nowadays beyond dispute. Two decades ago a committee of the American College of Physicians specifically supported the view that maintenance of a patient's functional well-being is a fundamental goal of medical practice. They also noted that the assessment of the physical, psychological, and social functioning of the patient in terms of the impact of disease is "an essential part of clinical diagnosis, a major determinant of therapeutic choices, a measure of their efficacy, and a guide in planning long-term care..."[1].

Since 2001, several impact high impact articles have been published on the effectiveness of HRQoL measurement in clinical practice, which have presented positive results such as more frequent discussion and identification of HRQoL related problems, improved emotional functioning, improved HRQoL, a decrease in depression, a decrease in debilitating symptoms, and expressed interest in continued use of the information by both physicians and patients [2-7]. Despite these positive results, standard measurement and feedback of HRQoL has as of yet not been widely implemented in clinical practice. This may be explained by the initial lack of convincing data regarding the effectiveness of standardized HRQoL measurement in actually improving HRQoL or psychosocial outcomes [3,8-11], and by practical and attitudinal barriers that have been associated with the implementation of HRQoL measurement in clinical practice. Practical barriers that have been reported include general lack of time, money and human resources, impracticality of instruments, disruption of clinical routine, lack of IT support and health professionals' lack of knowledge in this area. Attitudinal barriers may include health professionals' scepticism of the validity of HRQoL questionnaires, and ability to intervene should the questionnaires reveal any problems [11-17].

To the best of our knowledge, only two studies have actually implemented the procedure of HRQoL measurement in clinical practice and subsequently described the issues encountered in terms of feasibility. In one of the studies, the main finding was that higher compliance occurred when the computerized data collection was integrated into routine care. However, it should be noted that the follow-up time was very short (12 weeks), resulting in a large number of patients attending only once which makes it difficult to draw any firm conclusions on patient compliance in the long run [18]. In the other study, only 18 patients participated and the questionnaires were not computerized [19]. A previous study has shown that pen-and-paper versions of HRQoL questionnaires, which have to be scored by hand, take too much time and are costly in the long term [20]. Providing clinicians with instant information about their patients' HRQoL at busy outpatient clinics can only be obtained if this HRQoL is assessed by means of computers that can generate an output which can instantly be accessed by clinicians.

The aim of the present study was to gain more insight in the practical and attitudinal feasibility problems encountered during the process of implementing computerized HRQoL measurement at a busy outpatient department of Hepatology (liver disease) (Erasmus MC, Rotterdam, the Netherlands). Chronic liver disease is one of the most prevalent diseases in the world, affecting over 560 million people (, 4-12-2006). It is a serious disease that is associated with impaired HRQoL [21,22]. Chronic liver disease is an appropriate example of a typical chronic disease, with patients experiencing substantial comorbidity and possibly mortality as is the case in many other chronic diseases.

This study was among the first to actually implement the complete procedure of routine computerized HRQoL measurement at an outpatient department, and to subsequently describe all feasibility issues encountered throughout the process. The focus was on technical as well as logistic feasibility issues such as optimization of patient compliance in the long run, rather than effects of the intervention on patient well-being which have been presented elsewhere [3-7]. Practical suggestions for researchers and health care workers interested in implementing assessment of HRQoL in clinical practice were given.

## Methods

### Patient inclusion

This study was performed at the Department of Gastroenterology and Hepatology of the Erasmus Medical Centre (Rotterdam, the Netherlands), which is one of three specialised centres for liver disease in the Netherlands. With patients visiting the outpatient department on average once every four months, the recruitment phase was set at four months. Between September 2004 and January 2005 all patients of 18 years and older with chronic liver disease (CLD) attending the department of Hepatology, and all physicians working at the department of Hepatology, were invited to participate in the study verbally and in writing. Patients who agreed to participate received an explanation of the purpose and procedure of the study from the researcher, and consequently signed an informed consent form. The protocol was in accordance with the ethical guidelines of the modified 1975 Declaration of Helsinki and approved by the Medical Ethics Committee of the Erasmus MC.

### Study design and intervention

The first three months of the study consisted of a pilot-testing phase during which problems with the use of the computer program were detected and solved by asking patients to complete the online questionnaires. Patients' opinions on the computerized questionnaires were assessed by means of a pen-and-paper questionnaire that was administered afterwards. This questionnaire included the following questions: 'did you encounter any difficulties completing the questionnaires? If so, what?, 'what do you think of the time it took to complete the questionnaires?', and 'do you have any suggestions to improve the computer program? ' After these three months, the actual intervention started.

Physicians were randomly assigned to either the intervention group (who had access to a graphical representation of the HRQoL data of their patients) or the control group (who conducted their consultations as usual). The physicians in the intervention group were asked to use the HRQoL data in all consultations for the duration of one year. Physicians in both the control group and the intervention group were asked to complete a checklist about the content of the consultation after each consultation with a participating patient.

All participating patients were asked to complete computerized versions of a generic – (Short Form-12 [23]) and a disease-specific HRQoL questionnaire (Liver Disease Symptom Index 2.0 [24]), and the first part of a pen-and-paper questionnaire on patient satisfaction with the consultation, before each consultation (QUOTE-Liver [25]) for the duration of one year. After the consultation, they completed the second part of the satisfaction questionnaire. For a more elaborate description of the study design and intervention we refer to Gutteling et al. (2008)[7].

In order to optimise participation, study participants were given instructions on the study procedure both verbally and in writing at the beginning of the study, and eye-catching posters were put up in the waiting room to remind them of the study. In addition, the reception employees were instructed to refer study participants to the computer. With a study-duration of 1 year, it was estimated that this would yield on average three measurement moments per patient.

### Measurement instruments

#### Practical feasibility

Practical feasibility of computerized HRQoL measurement was assessed throughout the study by a) observing problems encountered during the development of the computer program which had to include several crucial specifications (instant scoring and graphical output of the data, instant availability of data to physicians, guaranteing patient privacy) b) by observing patients' ability to complete the HRQoL questionnaires and discussing any encountered difficulties. c) by monitoring the number of times that patients completed the HRQoL questionnaires. d) by observing logistics at the outpatient department on a daily basis. The observations were done by the main researcher on this project.

A questionnaire was administered retrospectively to assess participants' reasons for not completing the HRQoL assessments in the clinic. This questionnaire included the following questions: 1) Did you complete the questionnaires with each visit during the past year?, and 2) If not, please indicate why not. This last question had several response categories of which more than one could be checked: a) I forgot to complete the questionnaires, b) I was too late, or there was not enough time before the consultation to complete the questionnaires, c) I did not feel like completing the questionnaires, d) I was too ill to complete the questionnaires and e) other...

#### Attitudinal barriers

*Attitudinal barriers of physicians *were explored by semi-structured interviews with all physicians that were conducted midway through the study and at the end of the study. In these interviews physicians were asked, amongst others, whether they would be interested in continued use of the information and whether there were any items that they would like to be included in future versions of the computer program.

Secondly all physicians in the experimental group were asked to complete a checklist at the end of a consultation of each participating patient, which consisted of four important questions:, a) Did you request the HRQoL information?, b) Did you use the information? c) Did you find the information useful? and d) Why (not)?

*Attitudinal barriers on the part of the reception employees *were inventorized while observing the process of care at the outpatient department on a daily basis.

### Data analysis

The retrospective questionnaire administered to patients on reasons for not completing the assessment at the clinic and the checklist completed by physicians after each consultation were analysed quantitatively in SPSS 11.0, in terms of frequencies and percentages. Descriptive data is presented on the observed practical feasibility. Descriptive data on the interviews with physicians, which were intended to provide global information about physicians' experiences with, and opinions on, the HRQoL information, is also presented.

## Results

### Patients' and physicians' characteristics

**Figure 1:**
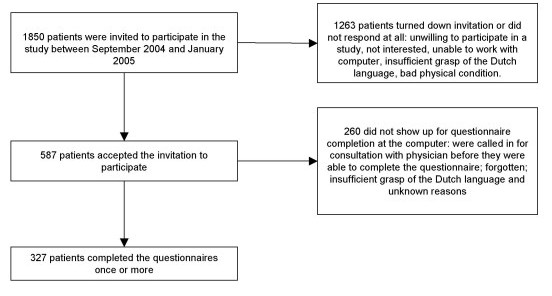
Patients in the study.

All physicians working at the department of Hepatology (n = 11, 10 = male, 1 = female) agreed to participate in the study. Their mean age was 39 years (range 27–55). The average working experience of the physicians was 8.7 years (range 0 – 27 years). Five hundred and eighty seven patients gave informed consent to participate (Figure 1) of which 327 completed the measurements once or more. 260 patients who had consented to participate did not complete the measurements once. Demographic characteristics of the 327 participants are presented in Table 1, and comparisons were made with the 260 non-responders.

**Table 1:** Demographic characteristics of patients in the study

	Respondents (n = 327)	Non-respondents (n = 260)	P
*Gender (n, %)*			
Women	144 (44)	108 (76)	0.46
Men	183 (56)	135 (42)	

*Age (mean, range)*	48.1 (20–81)	47.4 (18–80)	0.70

*Diagnosis (n, %)*			
Hepatitis B	47 (14)	43	0.00
Hepatitis C	47 (14)	54	
Cholestatic liver disease	33 (10)	31	
Pre-transplantation	18 (6)	1	
Post-transplantation	110 (34)	52	
Auto-immune hepatitis	23 (7)	16	
Other	49 (15)	47	

*Disease Severity (n,%)*			
No cirrhosis	206 (63)	153 (63)	0.95
Compensated cirrhosis	87 (27)	63 (26)	
Decompensated cirrhosis	34 (10)	28 (11)	

*Nationality (n, %)*			
Dutch	270 (83)	207 (85)	0.54
Moroccan	5 (2)	2 (1)	
Turkish	7 (2)	7 (3)	
Surinam	10 (3)	4 (2)	
Europe other	6 (2)	4 (2)	
World other	25 (7)	20 (7)	
Unknown	4 (1)	0 (0)	

Differences were assessed by means of Chi-square tests (except for age: t-test)

### Practical feasibility

#### Problems encountered during the development of the computer program

Developing a tailor-made computer program that met our needs with regard to the inclusion of our questionnaires of choice, lay-out, and instant availability of computerized graphical representations of the results to the physicians without violating patients' privacy, proved to be time-consuming and more costly than planned. Initially, we attempted to develop the computer program displaying the questionnaires with basic available computer programs (such as Microsoft Access). This eventually proved to be too complicated considering all the requirements outlined above that had to be met. Expertise of an IT professional was required. Finding an IT professional with the appropriate skills to develop the program cost several months as did the actual development of the final program. In terms of finances there are costs attached to the development of the program, the purchase of a domain name and airing the website where the questionnaires could be completed. During the pilot testing phase, we discovered that administering the Short Form-36, the complete LDSI 2.0 and the complete first part of the QUOTE-Liver interfered with clinical routine. Even though patients did not report negative evaluations regarding the length of the questionnaires, we included shorter versions of the questionnaires in the actual trial in order not to disrupt clinical routine [7]. Completion time was now on average 7.5 minutes, which we found acceptable since it did no longer interfere with clinical routine.

#### Patients' ability to complete the HRQoL questionnaires

During the pilot testing phase, problems with patients' basic computer skills such as mouse handling, scrolling and entering digits in a designated field became apparent. Some patients were not able to perform these skills. Although participants with such limited knowledge of computers formed a minority, they required substantial assistance. The computer program used in the trial was amended in order to overcome these problems by, as suggested by the patients participating in the pilot testing phase, making checkboxes larger and the entry field for the patient number more easily identifiable. Also, a mouse pad was used that provided step-by-step instructions for the completion of the questionnaires. These improvements did not visibly improve patient participation. The mouse pad was mostly ignored, and entering the patient number remained difficult, mostly because patients did not know their number (estimation of 1/2). Basic mouse handling also remained problematic for a significant amount of patients (estimation of 1/5), which consequently required substantial assistance.

#### HRQoL questionnaire completion rate

**Figure 2:**
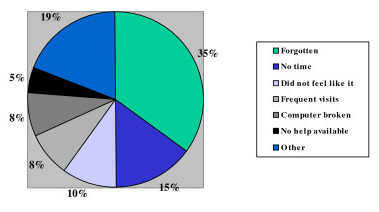
Participants' reasons for not completing the questionnaires.

At the end of the study, the HRQoL assessment in the clinic had occurred on 43% of the occasions (756 times out of the estimated 1761 times, which is a rough estimation based on the assumption that patients visited the outpatient department on average three times during the study (587 × 3 = 1761)). 260 participants never completed the HRQoL assessment on the computer at all, of which 16 due to substantial language problems. Only 105 patients completed the HRQoL questionnaires three times or more (Table 2). A retrospective exploration of the reasons for this low response rate was performed by means of a mailed questionnaire (response rate = 55%, 170 males, 145 females, mean age 50.0 years). The main reason that was given for not completing the retrospective questionnaires was 'simply forgetting'. Other important reasons included 'no time' and 'did not feel like it'. Less often, reasons such as 'the computer was broken', 'there was no-one there to help me complete the HRQoL questionnaires', 'no-one told me to complete the HRQoL questionnaires' and 'the computer was occupied', were given. For an overview of all reasons given we refer to Figure 2.

**Table 2:** Number of times that patients completed the questionnaires

	Times that questionnaires were completed									
	1	2	3	4	5	6	7	8	9	>9
Patients (n)	327	181	105	58	33	20	13	10	5	4

#### Logistical issues

Logistical issues that were observed at the outpatient department were forgetfulness of the reception employees to send patients to the computer, and the computer being out of sight of the waiting room area.

### Attitudinal barriers

#### Interviews with physicians

The interview data showed that all physicians would like to use the HRQoL information again in the future, especially for patients awaiting liver transplantation, patients with HCV, and non-native speakers (mostly patients with HBV). They suggested embedding the information in the existing patient information system, and adding a screening tool for depression, especially for patients with HCV and/or patients awaiting liver transplantation, diagnostic questions (e.g. allergies, use of medication), questions about the social situation of younger people (e.g. school, friends, pass-times), and questions about expectations of the consultation.

#### Physician checklists

**Figure 3:**
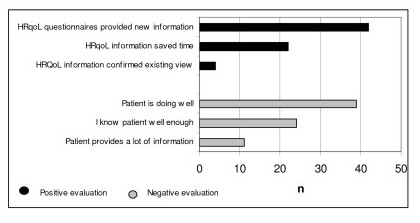
Physicians' evaluations of the HRQoL information.

The physicians in the experimental group requested the information in 92% of the consultations, discussing it with their patients in 58% of the consultations. They indicated finding the HRQoL information useful in 45% of the consultations, mostly because it provided new information, but also because it saved time and because it confirmed the verbal information and their own clinical impressions of patients who were doing well physically. These last two statements were also relevant for the one physician who claimed to know his patients well and did therefore not find the HRQoL information particularly useful. All physicians found the information less useful when patients were doing well, when they knew patients well and when patients were very talkative (Figure 3).

#### Observations

Attitudinal barriers were encountered on the part of the reception employees. Their busy schedule did not allow for much time to identify study participants and refer them to the computer. The importance to do so was not clear to them, and when no firm instructions were given, they often forgot to send patients to the computer.

#### Advice

The most important advice to improve HRQoL measurements in clinical practice that resulted from the current study is summarized in Table 3.

**Table 3:** Advice to improve HRQoL measurements in clinical practice

**Technical issues**
• For psychometric purposes, computerized questionnaires should resemble pen-and-paper versions as closely as possible
• Hire an IT expert
• Allow for development costs
**Logistical issues**
• Location in the vicinity of the waiting room area (ideally the computer can be seen from the waiting room area)
• Enough privacy
• Availability of internet/network connection
• Easily accessible to patients

**Optimal patient participation**
• use of a touch-screen computer
• very easy log-on procedure, eg. scanning the patient's punch card
• questionnaires in multiple languages
• short questionnaires
• HRQoL assessment is considered part of clinical routine
• Physicians and front desk employees ask patients to complete the questionnaires

**Optimal physician participation**
• HRQoL data embedded in the existing patient information system
• Add screening for depression
• Bring in a local clinical leader as a spokesman for the importance of HRQoL measurement
• Provide clear data output and clear instructions on how to interpret the data
• Make clear that the data should not be used as clear cut-off points for treatment of referral decisions, but rather as a base for more directed discussion of psychosocial topics
• Provide management options

## Discussion

The present study is, to the best of our knowledge, the first to describe a variety of feasibility issues encountered during the implementation of computerized HRQoL measurement in clinical practice, in a population of patients with chronic liver disease. Feasibility problems concerning technical aspects of developing a user-friendly computer program with safe data transmission over the Internet, patients' computer skills, and patients' compliance were encountered. Physicians were generally positive about the instant computerized availability of HRQoL information.

Technical problems that we encountered during the developmental phase of the computer program were substantial, and cost substantial time and effort to correct. Assistance from an IT professional is advised if one intends to develop a computer program that includes the particular questionnaires of interest, is easy for patients to complete, and transmits the information to the physicians' computer in such a way that privacy is assured.

With regard to patients' lack of basic computer skills, the use of touch-screen computers, which have been shown to be easy to handle by various patient populations [20,26-30], is recommended when implementing HRQoL measurement in clinical practice. This may optimise patient participation, and the quality of the answers, which will be less biased by the presence of family members or friends that help with completing the questionnaires such as found in the study of Velikova et al (2002) [31].

A limitation of the present study was the high number of non-participants. Part of the explanation may lie in the fact that patients themselves were responsible for contacting their physician if they were interested in participating in the study. In addition, the number of non-Dutch speaking patients visiting the department of Hepatology of the Erasmus MC is relatively large (Hepatitis B for example, is most common among people from North Africa). These patients were also invited to partcipate, but were not able to participate since the questionnaires in this study were only available in Dutch. Future studies should aim at including non-native speakers, whose data are of particular interest to the physicians in this study.

The low compliance of patients that did participate in our study, is in accordance with findings of a previous study showing deterioration of compliance with longer follow-up [17]. Bad timing and other priorities were given as possible explanations. In our study, an explanation may lie in the small window of opportunity to complete the questionnaires before each consultation. Indeed, patients mentioned in the retrospective questionnaire that lack of time was one of the main reasons for not completing the questionnaires. Simply forgetting to complete the questionnaires was the most important reason, despite eye-catching posters that were put up in the waiting room. The fact that the retrospective question "have you completed the questionnaires with each visit" was answered with "no" in 57% of the cases supposes an honest attitude of the respondents, who were informed about the anonymity of their responses. Considering these results, it seems that patient participation cannot be left to patients themselves, who may be nervous about the upcoming consultation and/or used to going to the waiting room after announcing themselves at the reception desk. To optimise participation it is, in our opinion, of vital importance that all staff of the department concerned, especially the reception desk personnel but also the nurses and physicians, approves of the intervention, considers it as part of standard clinical routine, and acts accordingly by sending patients to the computer before each consultation.

The positive attitudes of the physicians in our study towards the availability of instant computerized HRQoL information during the consultation are in accordance with previous studies in oncology [18,30], and advocate the continued use of such a procedure in patients with chronic liver disease. However, future studies should aim at including more liver specialists in order to substantiate these findings. Expressed concerns of an increase in workload as a result of the HRQoL data [30] were absent in our study. These positive findings in liver specialists, treating patients with a disease that is generally less acute and life threatening than cancer for instance, give incentive to further exploration of routine computerized HRQoL measurement in other specialisations within internal medicine such as nephrology or gastroenterology. When implementing such a procedure, it should be stressed to physicians that standardized HRQoL information should never replace the clinical dialogue between patient and physician, as important symptoms may then be overlooked, or exaggerated [30]. Rather, the HRQoL information should be seen as an indication of possible problems worth discussing and exploring further during the consultation.

## Conclusion

This study addressed practical feasibility issues associated with routine computerized measurement of HRQoL at a busy outpatient department of Hepatology. Feasibility is an important requirement for more widespread implementation of such an intervention. Another requirement is that the intervention is effective in improving patients' well-being and/or medical treatment. The current study has directly contributed to the first requirement by showing that the attitudes of physicians were generally positive, by identifying probable barriers towards successful implementation, and by providing solutions on how to overcome these barriers. These include hiring an IT expert, involving all personnel and using touch-screen computers. While the findings of the current study are encouraging they also emphasise that these implementation processes are complex and should not be underestimated. Further studying of the feasibility and effectiveness of routine computerized HRQoL measurements in clinical practice is needed before widespread implementation can be achieved.

## Competing interests

The authors declare that they have no competing interests.

## Authors' contributions

JG participated in the design of the study and conducted it. She also drafted the manuscript. RDM participated in the design of the study and helped to conduct it. JB and ASD participated in the design of the study and helped to draft the manuscript. All authors read and approved the final manuscript.
